# Systematic effects of patient factors and scanner/protocol factors on a Restriction Spectrum Imaging (RSI) quantitative MRI biomarker for prostate cancer

**DOI:** 10.1186/s40644-026-01032-w

**Published:** 2026-04-16

**Authors:** Deondre D. Do, Mariluz Rojo Domingo, Christopher C. Conlin, Ian Matthews, Karoline Kallis, Madison T. Baxter, Courtney Ollison, Yuze Song, George Xu, Allison Y. Zhong, Aditya Bagrodia, Tristan Barrett, Matthew Cooperberg, Felix Feng, Michael E. Hahn, Mukesh Harisinghani, Gary M. Hollenberg, Juan Javier-Desloges, Sophia C. Kamran, Christopher J. Kane, Dimitri Kessler, Joshua Kuperman, Kang-Lung Lee, Jonathan Levine, Michael A. Liss, Daniel J. A. Margolis, Paul M. Murphy, Nabih Nakrour, Michael A. Ohliger, Thomas Osinski, Anthony James Pamatmat, Isabella R. Pompa, Rebecca Rakow-Penner, Jacob L. Roberts, Karan Santhosh, Ahmed S. Shabaik, David Song, Clare M. Tempany, Shaun Trecarten, Natasha Wehrli, Eric P. Weinberg, Sean Woolen, Anders M. Dale, Tyler M. Seibert

**Affiliations:** 1 Department of Bioengineering, University of California San Diego, La Jolla, CA USA; 2 Department of Radiation Medicine, University of California San Diego, La Jolla, CA USA; 3 Department of Radiology, University of California San Diego, La Jolla, CA USA; 4 Department of Urology, University of California San Diego, La Jolla, CA USA; 5 Department of Radiology, University of Cambridge, Cambridge, UK; 6 Department of Urology, University of California San Francisco, San Francisco, CA USA; 7 Department of Radiation Oncology, University of California San Francisco, San Francisco, CA USA; 8 Department of Radiology, Massachusetts General Hospital, Boston, MA USA; 9 Department of Imaging Sciences, University of Rochester Medical Center, Rochester, NY USA; 10 Department of Radiation Oncology, Massachusetts General Hospital, Boston, MA USA; 11 Department of Urology, University of Texas Health Sciences Center San Antonio, San Antonio, TX USA; 12 Department of Radiology, Weill Cornell Medical College, New York, NY USA; 13 Department of Radiology and Biomedical Imaging, University of California San Francisco, San Francisco, CA USA; 14 Department of Urology, University of Rochester Medical Center, Rochester, NY USA; 15 Department of Computer Science, University of California San Diego, La Jolla, CA USA; 16 Department of Pathology, University of California San Diego, La Jolla, CA USA; 17 Department of Electrical and Computer Engineering, University of California San Diego, La Jolla, CA USA; 18 Department of Neurosciences, University of California San Diego, La Jolla, CA USA; 19 Halıcıoğlu Data Science Institute, University of California San Diego, La Jolla, CA USA

**Keywords:** Multiparametric magnetic resonance imaging (mpMRI), Clinically significant prostate cancer (csPCa), Apparent diffusion coefficient (ADC), Diffusion weighted imaging (DWI), Prostate Imaging Reporting & Data System (PI-RADS), Restriction spectrum imaging (RSI)

## Abstract

While multiparametric MRI has revolutionized clinically significant prostate cancer (csPCa) detection, conventional apparent diffusion coefficient (ADC) maps suffer from a monoexponential model that conflate the various forms of diffusion in the tissue, leading to poor microstructural specificity and substantial inter-scanner variability. Restriction Spectrum Imaging (RSI) addresses these limitations with a multi-compartment biophysical model that separates the diffusion signal into four compartments, providing a more specific quantitative imaging biomarker, the Restriction Spectrum Imaging restriction score (RSIrs). Although RSIrs has demonstrated promising detection performance for csPCa, patient- and acquisition-related factors may influence its measured values and performance. Our retrospective analysis included 1,890 men (median age 70) from seven centers between 2018 and 2024. Our primary objective was to characterize the quantitative technical robustness of RSIrs using linear modeling. We assessed how age, race, ethnicity, prostate volume, medication use, and MRI acquisition parameters influenced prostate maximum RSIrs (RSIrs_max_) values. Secondary, we conducted a functional validation of RSIrs’ csPCa detection performance when accounting for any significant effects (if any). Non-csPCa patients in this analysis were (1) with confirmed benign or Grade Group 1 findings on biopsy or (2) with a non-suspicious mpMRI (PI-RADS 1 or 2) and a prostate-specific antigen density (PSAD) ≤ 0.15. Results revealed modest effects from age (1.8/year increase), prostate volume (-0.83/mL decrease), and acquisition methods (varying between − 63.14 to + 56.23). While these acquisition- and patient-level variations partially overlapped with change in RSIrs_max_ due to Grade Group 2 csPCa, these variations were secondary to the primary signal variation due to csPCa. Adjusting for them did not improve csPCa detection performance (*p* > 0.05). AUC of RSIrs_max_ for patient-level csPCa detection was 0.77 [95% CI: 0.75–0.79] compared to 0.74 [0.72–0.76] after. RSIrs demonstrates robust csPCa detection performance regardless of modest signal variations from patient and acquisition factors, supporting its potential as a generalizable quantitative imaging biomarker for csPCa.

**Supplementary Information:**

The online version contains supplementary material available at 10.1186/s40644-026-01032-w.

## Introduction

Multiparametric magnetic resonance imaging (mpMRI) use in prostate cancer (PCa) treatment planning has greatly improved the detection and management of clinically significant PCa (csPCa: grade group [GG] ≥ 2), reducing unnecessary biopsies and overdiagnosis/overtreatment in men suspected of having csPCa [1]. Unfortunately, conventional mpMRI currently lacks a biomarker that accurately distinguishes between non-csPCa and csPCa without subjective expert radiologist interpretation. The apparent diffusion coefficient (ADC), a quantitative metric derived from diffusion-weighted imaging (DWI) during mpMRI, has shown poor reliability as a quantitative biomarker and is only clinically useful after identifying a suspicious lesion; by itself, it is inaccurate for patient-level detection of csPCa [4–6]. ADC’s model is too simplistic, providing only an ensemble average of diffusion properties within the complex microstructural environments of prostate tissue, limiting the specificity of ADC measurements [7]. For example, ADC values within tumors exhibit significant overlaps with non-malignant prostate conditions, including prostatitis and benign prostate hyperplasia [8, 9]. Variations in pulse sequences and *b*-values specific to individual clinical sites further impede the establishment of a standardized classification threshold for ADC to detect csPCa [10]. Despite these limitations, ADC is the current quantitative DWI metric used in the Prostate Imaging Reporting & Data System (PI-RADS) for detecting csPCa [11].

Restriction Spectrum Imaging (RSI) is a more sophisticated diffusion MRI approach that improves the qualitative [12] and quantitative [4, 13] interpretation of prostate MRI. Unlike conventional ADC, which provides a single average value of water displacement, Prostate RSI utilizes a multi-compartment biophysical model to decompose the diffusion-weighted MRI signal into four distinct microstructural environments: restricted intracellular, hindered extracellular, free diffusion, and vascular flow [4, 14]. Its accompanying biomarker, the RSI restriction score (RSIrs), isolates the restricted intracellular diffusion signal. This component targets the isotropic diffusion of water trapped within the high-density environment of tumor cells. Furthermore, RSI leverages the distinct T2-weighted properties of the intracellular space to account for the nuclear volume fraction; as tumors become more aggressive and the nuclear-to-cytoplasmic ratio increases, the RSIrs signal rises accordingly, providing a quantitative surrogate for histopathological Gleason Grade Group. At the whole-prostate level, the biomarker is applied by identifying the maximum voxel-wise value (RSIrs_max_) within the prostate gland. This approach is based on the clinical necessity of identifying the most aggressive focal region of a tumor, which determines patient prognosis. By utilizing the maximum value rather than a mean, the model captures the peak restricted signal of the index lesion and prevents signal dilution (i.e., compared to whole-gland averaging), at the cost of potential quantitative instability. This ensures that small but highly aggressive focal tumors are not obscured by the averaging effect of surrounding healthy or benign prostatic tissue, a common limitation when using whole-gland metrics.

RSIrs has been previously shown to outperform conventional ADC and perform comparably to expert PI-RADS interpretation [4, 13]. RSIrs maps accurately pinpoint csPCa and make it more noticeable to non-experts compared to mpMRI alone, facilitating improved precision of targeted cancer treatment [12, 14]. RSIrs provides objective estimates of the probability of csPCa without requiring expert radiologists [6] and has the potential to enhance the accuracy of csPCa detection in the early stages of PCa treatment planning, in conjunction with current clinical tools. There are known inaccuracies in tumor delineation [15] which could be improved with RSIrs. RSIrs provides objective estimates of the probability of csPCa, offering a standardized quantitative baseline that may augment expert interpretation. Rather than replacing the necessity of radiological expertise, this approach is intended to facilitate diagnostic consistency across centers and minimize the impact of reader variability. By providing a reproducible metric, RSIrs serves as a tool for clinical augmentation, potentially improving the efficiency and reliability of the mpMRI workflow.

However, a critical step for clinical implementation of any biomarker is understanding whether common patient or image acquisition factors may systematically bias RSIrs. Multicenter systematic bias in RSIrs has not been well quantified and represents a barrier for widespread clinical deployment. Therefore, the goal of this study is not to re-establish the diagnostic accuracy of RSIrs in isolation, but to determine whether the heterogeneity of clinical populations and real-world acquisition parameters can compromise its clinical utility.

Patient-level factors like age, prostate volume, and the use of 5-alpha-reductase inhibitors (5-ARIs) for treatment of benign prostate hyperplasia (e.g., finasteride and dutasteride) can impact interpretation of mpMRI [16, 17]. Racial and ethnic disparities are well documented for PCa, particularly among Black or African American men, although mpMRI appears equally useful for patients of different races and ethnicities with expert interpretation [18, 19]. It was previously found that changing the echo time during RSI acquisition has a modest effect on RSIrs that can be effectively accounted for through simple calibration [20]. However, differences in RSIrs resulting from variation in scanner model/manufacturer and other acquisition parameters are not well understood.

The primary objective of this study was to quantify the technical robustness and multicenter reproducibility of the RSIrs biomarker across an ensemble of scanner platforms, acquisition protocols, and patient populations.

As a secondary objective, we evaluated the performance of RSIrs for detecting csPCa before and after accounting for any observed technical variability to investigate any substantial effects on csPCa detection performance.

## Methods

### Population demographics

Prostate MRI data from 2845 patients with RSI were retrospectively collected from seven imaging centers belonging to the Quantitative Prostate Imaging Consortium (QPIC); collected scans ranged from 2018 to 2024. These institutions include the Center for Translational Imaging and Precision Medicine at the University of California San Diego (UCSD CTIPM), Massachusetts General Hospital (MGH) affiliated with Harvard University, UC San Diego Health (UCSD Health), University of California San Francisco (UCSF), University of Rochester Medical Center (URMC), University of Texas Health Sciences Center San Antonio (UTHSCSA), and University of Cambridge. A subset of 151 participants were included in a previous preliminary, single-center study [4]. All previous investigations of RSI were conducted using only a single MRI scanner. All participating institutions received approval from their respective institutional review boards (IRBs). Prospectively gathered data from UTHSCSA and Cambridge were collected with written informed consent from participants, while the remaining institutions obtained a consent waiver from their IRBs for retrospective use of clinical records. All research was conducted in accordance with the relevant guidelines and regulations set in place by each institutions’ IRB. Clinical records were reviewed to extract PI-RADS scores, biopsy results, and patient-level factors of interest, including age, race, ethnicity, prostate volume, and 5-ARI use.

Male patients over 18 years old who underwent prostate mpMRI with RSI were eligible for inclusion in this study. Exclusion criteria included prior treatment for PCa, metal implants in the pelvis, and lack of available biopsy results within 6 months of a positive mpMRI (PI-RADS ≥ 3). Non-csPCa patients were defined as those (1) with confirmed benign findings or GG 1 PCa based on biopsy histopathology, or (2) with a non-suspicious mpMRI (PI-RADS 1 or 2) and a prostate-specific antigen density (PSAD) ≤ 0.15. Patients with mpMRI and a PSAD ≥ 0.15 without available biopsy results were excluded. Patients were categorized into acquisition groups based on scanner model/manufacturer and RSI protocol (Supplementary Table 1). Acquisition groups were formed based on the following criteria: same scanner model/manufacturer, equivalent *b*-values, similar voxel size (within 25%), similar TE (within 10 ms). Any groups with less than 15 patients were excluded. All scans were obtained using 3-tesla MRI scanners (GE Discovery MR750/SIGNA Premier or Siemens MAGNETOM Skyra/Trio) from two manufacturers (GE Healthcare, Waukesha, WI, USA; SIEMENS Healthineers, Erlangen, Germany). Clinical MRI examinations were performed and interpreted following the guidelines of PI-RADS v2/v2.1. All MRI data was anonymized prior to analysis. Prostate segmentation was conducted using an FDA-cleared artificial intelligence tool (OnQ™ Prostate - Cortechs.ai; San Diego, CA) which has been previously validated to yield results comparable to manual segmentation by an expert radiation oncologist [21].

### RSI data acquisition and processing

All post-processing was done using MATLAB (MathWorks; Natick, MA) with custom programs, including correction of distortions induced by *B*_*0*_ inhomogeneity, eddy currents, and gradient nonlinearity [7, 22]. Noise correction was performed to eliminate bias in the DWI signal stemming from the presence of the noise floor. Linear fitting of the RSI model (Eq. 1) to the post-processed DWI data was performed to estimate signal contributions from each of the four RSI model compartments (C_1_: restricted intracellular, C_2_: hindered extracellular, C_3_: free diffusion and C_4_: vascular flow). The RSI-C_1_ compartment signal was normalized by the median DWI signal at b = 0 (s/mm^2^) within the prostate to calculate each patient’s voxel-wise RSIrs map.1

Equation 1: Formula for computing RSIrs where S(*b*) represents the RSI signal based on the *b*-value from diffusion weighted imaging (arbitrary signal units). C_i_ denotes the RSI compartment signal contribution, and D_i_ is the fixed compartmental diffusion coefficient as described by Conlin et al. [14] mb0 is the median DWI signal at b = 0 (s/mm^2^) within the prostate.

### Primary objective: assessment of technical robustness and variability

The primary analysis evaluated systematic effects of acquisition- and patient-level factors on RSIrs using linear modeling. Factors assessed included age, race/ethnicity, prostate volume, 5-ARI use (defined as currently taking this medication or medication use ended within 6 months before MRI), and acquisition group (representing MRI scanner manufacturer/model and RSI acquisition parameters). For categorical variables, the reference groups were as follows: White Non-Hispanic for race/ethnicity group, no current/recent 5-ARI use, and a common acquisition group at the two UCSD centers (denoted UCSD CTIPM_Discovery1_/UCSDH_Discovery_). We included cancer GG in the model due to the strong association of RSIrs with GG, which is the primary rationale for its utility as a csPCa biomarker. GG was included as a categorical variable with benign as the reference and GG 1, 2, 3, or 4–5 as other values [4, 6, 23, 24]. We modeled the effects of these parameters on the maximum RSIrs (RSIrs_max_) in the prostate, as it is a patient-level detector of csPCa and the most studied application of RSIrs [4, 6]. Since there was only one RSIrs_max_ value per patient, we employed a multiple linear regression model using fitlm() in MATLAB [25]. We repeated this analysis using a separate statistical (linear mixed effects) model using all prostate voxels in strictly patients without csPCa, to avoid any cancer-related effects on RSIrs (Supplementary Eq. 1) [26].

To ensure the validity and stability of our statistical model estimates, we implemented a diagnostic pipeline to ensure the assumptions of linear regression were met. Multicollinearity among fixed effects was assessed using Variance Inflation Factors (VIF) on the final analytic cohort (*n* = 1,050). Model assumptions, including normality and homoscedasticity of residuals, were verified through the examination of Pearson residuals via Q-Q plots and residual-vs-fitted plots. Furthermore, influential observations were screened by: (1) identifying outliers (standardized Pearson residual > | 3 |) and high-leverage points (*l* > 0.022). To confirm that the model was not disproportionately driven by individual cases, we evaluated the intersection of these metrics to identify points that were simultaneously high-leverage and high-outlier.2

Equation 2. Multiple linear regression model formula to predict maximum RSIrs (RSIrs_max_) within the prostate for a given patient based on patient and acquisition factors. β_0_, β_1_, …, β_5_, β_6_ denotes the respective predictor coefficient estimates, *i* represents the *i-*th patient, and ε represents the vector of residual error terms. 

To separate patient- from acquisition-related variability, we repeated this analysis using a linear mixed-effects model with a hierarchical approach, depicted in Eq. 3 below. Patient-level covariates (age, 5-ARI use, prostate volume, race/ethnicity, and Grade Group) remained included as fixed effects to control for patient case-mix. Imaging center, scanner manufacturer/model, and acquisition protocol were instead modeled as random intercepts. Random effects were specified as crossed effects, as scanner models and protocols were shared across multiple centers. Variance components were calculated to quantify the proportion of total RSIrs variability attributable to each acquisition-level factor. Intraclass correlation coefficients (ICCs) were calculated for clustering effects. 

Equation 3. Linear mixed-effects model formula to predict maximum RSIrs (RSIrs_max_) within the prostate for a given patient based on patient-level and acquisition-level factors. *β*_0_, *β*_1_, …, *β*_4_ denote the fixed-effect coefficient estimates; *u*_j_, *v*_k_, and *w*_l_ represent the random intercepts for MRI imaging center (*j*), manufacturer model (*k*), and acquisition protocol (*l*), respectively; *i* represents the *i*-th patient; andϵ_*i*_represents the residual error term.3

All multivariable regression and hierarchical mixed-effects models were performed using complete cases. Acquisition parameters, imaging center information, and pathological outcomes were available for the full study cohort (*N* = 1,890). 59 subjects (3.1%) had non-standard acquisition parameters that did not fall within predefined acquisition groups and were excluded from analyses involving acquisition-group modeling. Among patient-level covariates, 4 subjects (0.2%) were missing age, 499 (26.4%) were missing Grade Group (i.e., due to no biopsy/pathology data), 92 (4.87%) were missing prostate volume, and 333 (17.6%) were missing race/ethnicity; these cases were excluded from all statistical estimation models (*n* = 1050). There was no evidence of differential missingness by csPCa status. Diagnostic performance analyses were conducted in the full cohort, as outcome data were complete for all patients.

### Secondary objective: functional diagnostic validation

As a secondary analysis, we assessed whether adjustments for acquisition- and patient-level variations influenced the csPCa detection performance of RSIrs_max_. Adjusted RSIrs_max_ values were derived using estimated linear shifts from the primary model. We then computed the area under the receiver operating characteristic (ROC) curve (AUC) before and after adjustment to assess the impact of these effects. To account for the uneven distribution of patient and acquisition factors in our dataset, we randomly sampled from a subgroup consisting of only patients with the factors of interest and matched them with patients without those factors but whose cancer was of the same Grade Group (GG). We stratified our random sampling based on GG to mitigate effects on AUC due to varying proportions of csPCa. Median differences in AUC and 95% confidence intervals (based on 10,000 bootstrap samples) were compared before and after adjustment for patient and acquisition factor effects. Secondarily, we also re-ran the matching and bootstrapping within subgroups selected only for the statistically significant factors and compared pre- and post-adjustment within these distinct subgroups.

### Sensitivity analysis: assessment of performance stability

In this study, csPCa was defined as Grade Group ≥ 2 on histopathology. Patients with PI-RADS 1–2 and PSA density ≤ 0.15 ng/mL^2^ who did not undergo biopsy were classified as non-csPCa, reflecting current clinical practice in which biopsy is often deferred in this low-risk population. To evaluate potential verification bias arising from this composite reference standard, sensitivity analyses were performed in multiple sub-cohorts: (1) a biopsy-confirmed cohort (*n* = 1,392), excluding all proxy negatives; (2) patients undergoing systematic biopsy only (*n* = 343); and (3) patients undergoing both systematic and targeted biopsy (*n* = 709). Furthermore, to mitigate potential bias from post-biopsy hemorrhage and inflammation (which can induce susceptibility artifacts and signal attenuation) and other biopsy-related morbidities, we repeated this analysis on a (4) biopsy-naïve sub-cohort (*n* = 1,233). Patients were included in this sub-analysis only if their MRI scan preceded their biopsy date or if they had no prior history of prostate biopsy. Model estimates and diagnostic performance were recalculated in each subgroup to assess for substantial changes to the results in our analyses.

### Clinical reliability: sample estimation of significant effects

It is unknown how many patients are needed to estimate systematic effects on RSIrs_max_ due to patient or acquisition factors. This could influence interpretation of the present study’s statistical power, and it would be helpful to know how many patients might be needed to estimate additional possible effects in the future. To determine the minimum number of patients needed to estimate a patient or acquisition effect, we analyzed patient subgroups with a given factor and compared them to patients from the reference population. We iteratively bootstrapped 10,000 samples, from a sample of size one to the maximum group size. For each of these 10,000 bootstrap groups, we calculated the median RSIrs effect coefficient estimate for the factor of interest and the corresponding 95% CI. The minimum sample size to yield a bootstrap median effect estimate within 1% of the total population coefficient estimate was considered a reasonable threshold for the minimum sample size to estimate acquisition group effects.

## Results

**Fig. 1:**
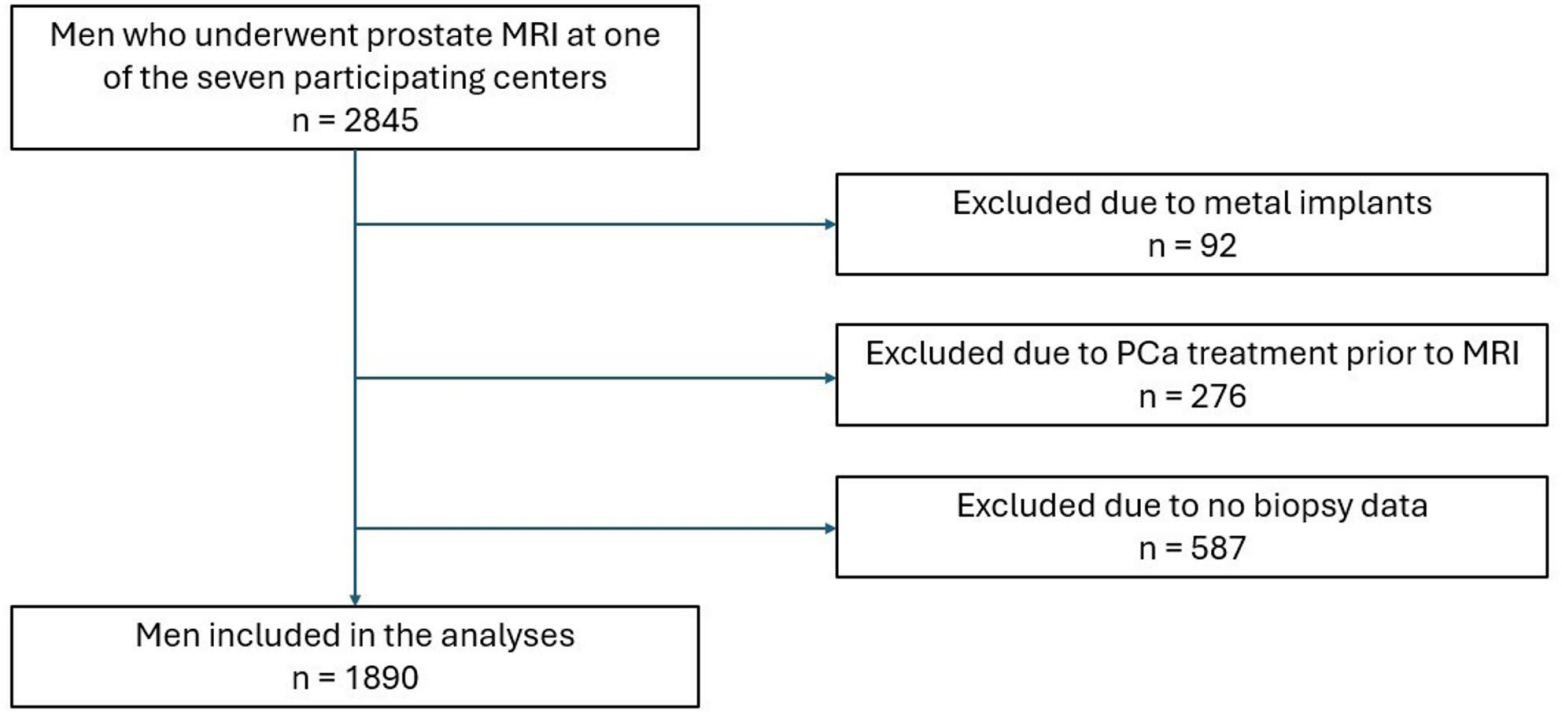
Patient flowchart depicting the selection process of 2845 men over 18 years old undergoing prostate MRI. After applying exclusion criteria, 1890 men were included in the final analysis. 1 additional patient from this dataset was excluded from analysis due to artifact that yielded RSIrs_max_ greater than 15 standard deviations from the population mean

1890 patients met the inclusion criteria (Fig. 1). Among them, 94 patients self-identified as White and Hispanic, 1226 as White and Non-Hispanic, 65 as White and Other/Unknown ethnicity, 120 as Asian, 117 as Black, 6 as American Native, and 6 as Native Hawaiian or Other Pacific Islander; race was not reported by 256 patients. 84 patients were currently on 5-ARIs at the time of MRI or had used the medication in the 6 months before their prostate MRI. The median age was 70 with an interquartile range (IQR) of 64–75 years. The median prostate volume was 51 with an IQR of 36–74 mL. One outlier was excluded due to artifact that yielded RSIrs_max_ greater than 15 standard deviations from the population mean (Table 1).

**Table 1:** Patient characteristics

Patient Characteristics, Total Study Participants (*n* = 1890)	
**Patient Cohorts**	
UC San Diego Health	692
UC San Diego CTIPM	688
Harvard University Massachusetts General Hospital	64
University of Rochester Medical Center	251
UC San Francisco	43
UT Health Sciences Center San Antonio	147
University of Cambridge	5
**Clinical Parameters**	
Age (years), median [IQR]	70
	[64,75]
Prostate volume (ml), median [IQR]	51
	[36–74]
**PSA**	
PSA density ≤ 0.15	1094
PSA density > 0.15	796
**Biopsy**	
Receive Biopsy Prior to MRI scan	657
Biopsy-naïve at time of MRI scan (had a biopsy within 6 months after MRI)	1233
**Pathology**	
Systematic biopsy only	503
Targeted biopsy only	179
Systematic and targeted biopsy	709
Prostatectomy	323
No biopsy within 6 months of MRI scan	499
**PIRADS (v2/v2.1)**	
1	635
2	53
3	263
4	453
5	442
Unavailable	44
**Gleason Grade Group**	
No Available Pathology	499
Benign	334
1	296
2	367
3	211
4	81
5	102
**Race & Ethnicity**	
White, Hispanic	94
White, Non-Hispanic	1226
White, Ethnicity Other/Unknown	65
Asian	120
Black	117
American Indian/Alaska Native	6
Native Hawaiian or Other Pacific Islander	6
Other / Unknown	256
**5-Alpha-Reductase Inhibitor (5-ARI) Usage**	
5-ARIs currently prescribed during MRI scan	84
5-ARIs previously taken (> 6 months before MRI/Date Unknown)	143
**Scanner/Protocol Group Cohorts**	
Cambridge	5
MGH	63
UCSD CTIPM_Discovery1_/UCSDH_Discovery_	844
UCSD CTIPM_Discovery2_	167
UCSDH_Premier_	143
UCSF/UCSD CTIPM_Premier_	220
UTSA_Skyra_	55
URMC	251
UTSA_Trio_	88
Other	54
**Scanner Manufacturer & Model**	
GE Discovery MR750 and MR750W (3T)	1058
Siemens Skyra (3T)	310
GE SIGNA Premier (3T)	434
Siemens Tim Trio (3T)	88

Abbreviations: PSA (Prostate Specific Antigen), Cambridge (University of Cambridge), MGH (Harvard University’s Massachusetts General Hospital), UCSD[H] (University of California San Diego [Health]), CTIPM (Center for Translational Imaging and Precision Medicine), UCSF (University of California San Francisco), URMC (University of Rochester Medical Center), UTHSCSA (University of Texas Health Sciences Center San Antonio)

Multicollinearity in our models was negligible, with all predictors yielding VIF values < 1.7 (range: 1.01–1.66), below the standard conservative threshold of 5.0. Residual analysis indicated that the model assumptions were satisfied (Supplementary Fig. 3A/B/D). While 11 outliers and 82 high-leverage points were identified, zero observations were found to be simultaneously high-leverage and high-outlier, confirming that the model estimates are stable and represent the full multicenter cohort (Supplementary Fig. 3C).

Statistically significant effects (*p* < 0.05) on RSIrs were observed for age (1.81/year), prostate volume (-0.83/mL) and three acquisition groups (UCSD CTIPM_Discovery2_[-52.21], URMC[56.23], and UTSA_Trio_[-63.14]). Detailed fixed-effect coefficients with 95% confidence intervals and coefficient-level partial R² values of significant effects are presented in Table 2. Grade Group 5 was the strongest predictor of RSIrs values in the full patient cohort (i.e., coefficient-level partial R² = 0.093). Acquisition-related factors demonstrated comparatively small effect sizes (coefficient-level partial R² for any factor did not exceed 0.012). These results were reflected in the global partial R^2^ values (Grade Group: 0.111; Acquisition Group: 0.034). Additional global partial R^2^ values are shown in Supplementary Table 8.

**Table 2:** All predictors and their estimated effects on the RSIrs biomarker identified using multiple linear regression modeling

Model	All Patients (RSIrs_max_) [*n* = 1890]	Biopsy-Naïve [*n* = 1233]	Biopsy-Confirmed [*n* = 1392]	Systematic Biopsy Only [*n* = 343]	Targeted + Systematic Biopsy [*n* = 709]
**Formula**					
	RSIrs_max_ ~ 5-ARI use + Age + Prostate volume + Race/Ethnicity group + Grade group + Acquisition group	RSIrs_max_ ~ 5-ARI use + Age + Prostate volume + Race/Ethnicity group + Grade group + Acquisition group	RSIrs_max_ ~ 5-ARI use + Age + Prostate volume + Race/Ethnicity group + Grade group + Acquisition group	RSIrs_max_ ~ 5-ARI use + Age + Prostate volume + Race/Ethnicity group + Grade group + Acquisition group	RSIrs_max_ ~ 5-ARI use + Age + Prostate volume + Race/Ethnicity group + Grade group + Acquisition group
**Age** [reference = mean, 69 years old]					
	1.81* **[0.23**,** 3.38] ||** R² = **0.005**	2.36* **[0.20**,** 4.51] ||** R² = **0.007**	1.81* **[0.23**,** 3.38] ||** R² = **0.005**	0.25 [-2.24, 2.74]	1.75 [ -0.82, 4.30]
**Prostate Volume** [reference = mean, 60 mL]					
	-0.83*** **[-1.25**,** -0.41] ||** R² = **0.012**	-0.89* **[-1.46**,** -0.32] ||** R² = **0.014**	-0.83*** **[-1.25**,** -0.41] ||** R² = **0.012**	-0.52 [-1.28, 0.24]	-0.76* **[-1.43**,** -0.08] ||** R² = **0.009**
**5-ARI Use** [reference = Not used]					
On Use within 6 months of MRI	36.40 [-32.47, 105.26]	55.77 [-39.81, 151.34]	36.40 [-32.47, 105.26]	22.43 [-76.91, 121.77]	48.73 [-53.85, 151.32]
**Race/Ethnicity Group** [reference = White Non-Hispanic]					
Asian	17.84 [-33.58, 69.27]	35.32 [-31.97, 102.62]	17.84 [-33.58, 69.27]	-7.04 [-76.28, 62.20]	52.30 [-27.79, 132.39]
Black	26.84 [-16.71, 70.38]	-11.22 [-75.94, 53.51]	26.84 [-16.71, 70.38]	59.71 [-8.66, 128.07]	20.39 [-54.09, 94.87]
White Hispanic	6.71 [-43.9, 57.32]	-44.33 [-127.42, 38.77]	6.71 [-43.9, 57.32]	52.67 [-21.50, 126.84]	-17.34 [-90.97, 56.30]
**Grade Group** [reference = Benign]					
1	17.03 [-17.79, 51.85]	16.98 [-32.25, 66.20]	17.03 [-17.79, 51.85]	-15.69 [-71.71, 40.33]	37.55 [-15.61, 90.71]
2	61.01*** **[26.79**,** 95.23] ||** R² = **0.012**	60.79** **[14.64**,** 106.94] ||** R² = **0.010**	61.01*** **[26.79**,** 95.23] ||** R² = **0.012**	43.74 [-13.23, 100.71]	82.07** **[28.37**,** 135.77] ||** R² = **0.016**
3	101.89*** **[61.92**,** 141.87] ||** R² = **0.024**	93.31*** **[38.891**,** 147.73] || R² = 0.017**	101.89*** **[61.92**,** 141.87] ||** R² = **0.024**	116.43*** **[54.04**,** 178.82] ||** R² = **0.055**	121.54*** **[53.93**,** 189.15] ||** R² = **0.022**
4	158.04*** **[96.97**,** 219.12] ||** R² = **0.024**	155.90*** **[65.91**,** 245.88] ||** R² = **0.017**	158.04*** **[96.97**,** 219.12] ||** R² = **0.024**	160.92*** **[71.409**,** 250.43] ||** R² = **0.051**	144.25*** **[49.86**,** 238.64] ||** R² = **0.016**
5	266.75*** **[215.73**,** 317.76] ||** R² = **0.093**	275*** **[205.35**,** 344.64] ||** R² = **0.083**	266.75*** **[215.73**,** 317.76] ||** R² = **0.093**	240.35*** **[166.47**,** 314.23] || R² = 0.15**	387.34*** **[293.09**,** 481.58] ||** R² = **0.105**
**Acquisition Group** [reference = UCSD CTIPM_Discovery1_/UCSDH_Discovery_]					
MGH	7.90 [-94.5, 110.33]	-28.11 [-190.36, 134.14]	7.90 [-94.5, 110.33]	54.32 [-43.872, 152.52]	22.75 [-247.05, 201.56]
UCSD CTIPM_Discovery2_	-52.21* **[-98.11**,** -3.26] ||** R² = **0.005**	-70.61* **[-139.34**,** -1.87] ||** R² = **0.006**	-52.21* **[-98.11**,** -3.26] ||** R² = **0.005**	-58.11 [-117.49. 1.27]	-34.39 [-109.96, 41.18]
UCSDH_Premier_	33.99 [-13.1, 81.10]	12.55 [-44.44. 69.54]	33.99 [-13.1, 81.10]	81.6* **[6.4801**,** 156.71] ||** R² = **0.02**	18.30 [-49.28, 85.87]
UCSF/UCSD CTIPM_Premier_	-39.08 [-86.39, 8.23]	-54.23 [-118.73, 10.27]	-39.08 [-86.39, 8.23]	54.36 [-36.89, 145.62]	-64.71 [-132.65, 3.23]
URMC	56.23*** **[24.50**,** 87.95] ||** R² = **0.012**	40.69* **[0.80**,** 80.59] ||** R² = **0.006**	56.23*** **[24.50**,** 87.95] ||** R² = **0.012**	12.42 [-103.86, 128.69]	66.39* **[5.14**,** 127.64] ||** R² = **0.008**
UTSA_Skyra_	-8.25 [-75.24, 58.74]	-5.89 [-121.84, 110.05]	-8.25 [-75.24, 58.74]	54.02 [-61.33, 169.38]	-22.40 [-119.44, 74.64]
UTSA_Trio_	-63.14* **[-111.56**,** -14.72] ||** R² = **0.006**	-	-63.14* **[-111.56**,** -14.72] ||** R² = **0.006**	-75.90* **[-140.44**,** -11.33] ||** R² = **0.02**	-43.75 [-116.40, 28.91]

These predictors included 5-ARI (current 5-ARI usage or usage < 6 months before MRI), age, prostate volume, race/ethnicity, grade group, and acquisition group. Coefficient estimates [95% confidence interval] are reported for each significant effect, which provide insight into the impact of these variables on RSIrs. Intraclass correlation coefficients (R^2^) are reported for each significant effect. *N/A* signifies there were no representative patients of that category included in the analysis. Significant predictors: * (*p < 0.05)*, ** (p < 0.01), *** (p < 0.001)

**Fig. 2:**
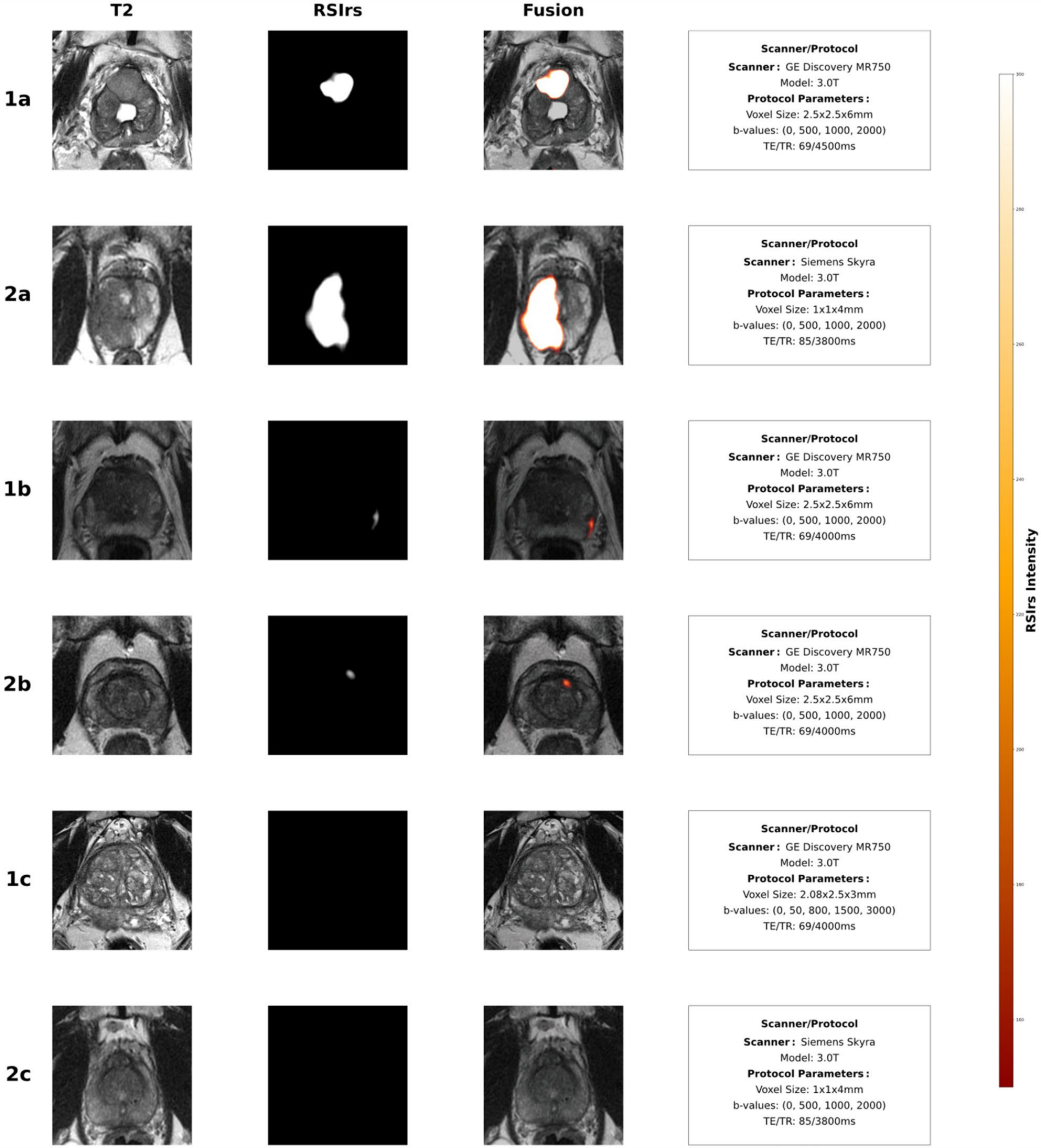
Axial T2-Weighted (T2W) images, RSIrs maps, and RSIrs overlaid on T2W imaging for six male patients, illustrating consistent imaging for detection of csPCa despite differences in patient and acquisition factors. These images illustrate the similarities in RSIrs_max_ regardless of patient and acquisition factors. Arrows indicate the prostate at the slice location of clinically determined prostate lesions, except for Patients 1c and 2c, who had no clinically significant lesions (PI-RADS > 3). All patients with clinically significant prostate cancer (csPCa) (GG > 2) were confirmed by targeted biopsy. Details regarding specific acquisition-level factors are listed in annotation boxes to the right of RSIrs overlay fusion images. The patients are categorized into three groups based on grade group: **High-Risk (Patients 1a and 2a)**: Biopsy-proven GG5, with a PI-RADS score of 5, from two different institutions: UCSD and URMC respectively. RSIrs_max_ was > 500 for both these patients. UCSD imaging was obtained with a GE scanner, and the URMC imaging was obtained with a Siemens scanner. **Intermediate (Patients 1b and 2b)**: Biopsy-proven GG3, with a large age difference (over 15 years). RSIrs_max_ was between 300 and 350 for both these patients. Patient 1b had a PI-RADS score of 3, and Patient 2b had a PI-RADS score of 5. **Non-csPCa (Patients 1c and 2c)**: These patients have a large discrepancy in prostate size (22 and 129 cubic centimeters, respectively), both with a PI-RADS score of 1. RSIrs_max_ was < 200 for both these patients. Patient 1c was biopsy-proven benign, and Patient 2c was biopsy-proven GG1

In our diagnostic performance analyses, the unadjusted AUC for detection of clinically significant prostate cancer was 0.77 (95% CI: 0.75–0.79). Following adjustment for significant factors, the AUC was 0.74 (95% CI: 0.72-0.076). Center-level analyses (Supplementary Table 7) demonstrated consistent unadjusted csPCa detection performance across nine acquisition groups, with AUC values ranging from 0.73 to 0.81 despite variation in sample size and cancer prevalence, except UCSF whose population’s AUC was 0.58 (95% CI:0.37,0.78). As expected, GG ≥ 2 had a large, significant statistical effect on RSIrs_max_ (median: 61.01,266.75 RSIrs). We repeated the analysis using the 99th percentile RSIrs due to presumed decreased variability/noise compared to the 100th percentile (RSIrs_max_) and obtained similar results (Supplementary Table 2); we repeated the analysis using a linear mixed effects model with patients without csPCa and found additional significant effects by acquisition group (UCSF/UCSD CTIPM_Premier_, UCSDH_Premier_, and UTSA_Skyra_), none were larger than 20 RSIrs (Supplementary Table 3). RSIrs maps similarly had consistent imaging despite significant differences in patient and acquisition effects (Fig. 2).

Linear mixed-effects modeling with our hierarchical model demonstrated that acquisition-related factors contributed modestly to overall RSIrs variability. Imaging center accounted for 9.23% of total variance (ICC = 0.0923), scanner manufacturer/model 3.44%, and acquisition protocol 4.88%, for a combined acquisition-level contribution of 17.1% (Supplementary Table 6). The remaining 82.45% of variance was attributable to patient-level and residual factors. Grade Group was the strongest fixed-effect for RSIrs; for example, Grade Group 5 was associated with an increase of + 259.75 units (95% CI: [210.64, 308.87]; *p* < 0.001), magnitudes higher than the standard deviation associated with any individual acquisition-related effect (Imaging Center: 62.76, Manufacturer Model: 38.30, Protocol: 45.65).

**Fig. 3:**
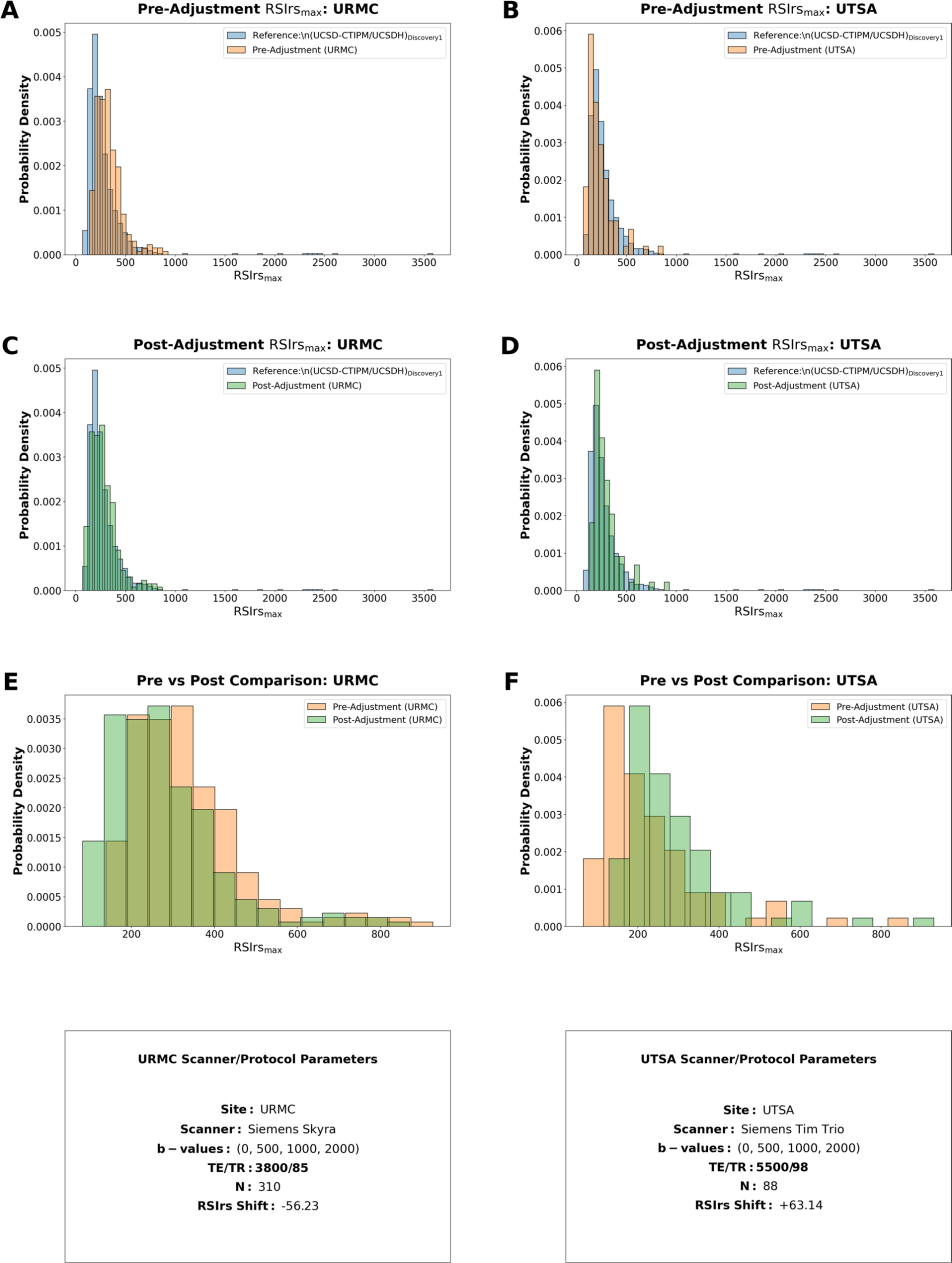
Histograms comparing the pre-adjustment and post-adjustment RSIrs distributions for two institutions with significant acquisition effects, URMC and UTSA_Trio_ (respectively in each, the orange and green histograms), and the reference group UCSD CTIPM_Discovery1_/UCSDH_Discovery_ (blue histogram) are shown in plots (**A**/**B**) and (**C**/**D**). Plot (**E**) and (**F**) show the pre- and post-adjustment RSIrs within each acquisition group. Details regarding the specific acquisition factors are displayed below Plots E/F. The distributions are significantly different (*p < 0.05)* before and after adjustment with Mann-Whitney U testing

**Fig. 4:**
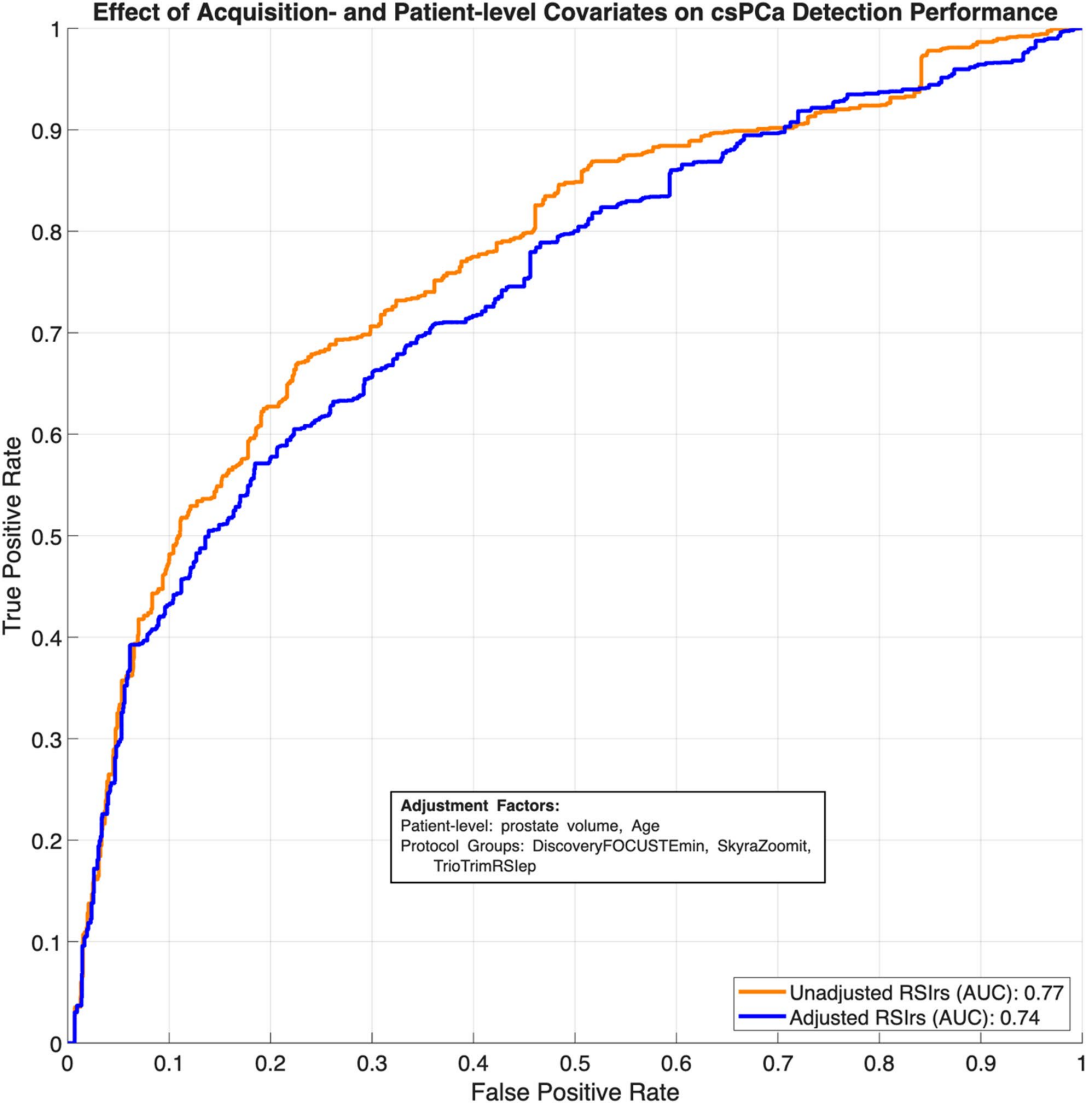
ROC curves illustrate the detection performance of RSIrs_max_ for clinically significant prostate cancer (csPCa) pre-adjustment and post-adjustment using shifts estimated from all patients. Pre-adjustment RSIrs_max_ performance (orange line) is compared with post-adjustment performance (blue line) after pooling data from 10,000 bootstrap samples. The area under the curve (AUC) values are reported for each model, demonstrating the impact of acquisition and patient adjustments on the predictive accuracy of RSIrs_max_ for csPCa. Median pre-adjustment AUC was 0.77 [95% CI: 0.75–0.79] and median post-adjustment AUC was 0.74 [0.72–0.76]. Adjustment for patient and acquisition effects does not significantly affect the AUC (*p* > 0.05)

Considered alone, adjustment appears to visually increase overlap between RSIrs distributions of effect groups to the reference, but this effect was not statistically significant (*p* ≥ 0.05) (Fig. 3). Adjustment for patient and acquisition factors did not improve detection of csPCa with RSIrs (*p* ≥ 0.05). Pre-adjustment AUC was 0.77 [95%CI: 0.75–0.79] and post-adjustment AUC was 0.74 [95% CI: 0.72–0.76] (Table 3; Fig. 4). Repeating this analysis using only patients without csPCa returned similar results (Supplementary Table 4; Supplementary Fig. 1). A secondary analysis using patient subgroups selected specifically for the statistically significant factors similarly revealed no improvement of AUC when adjusting for factors using all patients or only those without csPCa (Supplementary Table 5). Furthermore, following the most conservative statistical adjustment (Method 2), the performance remained stable with an AUC of 0.71 (95% CI: 0.68–0.72), mirroring the trends observed in the full multicenter cohort.

**Table 3:** Results from a 10,000-bootstrap analysis using a subgroup of patients with significant acquisition and patient effects on RSIrs_max_

Cohort	Sample Size (*n*)	Median AUC (Pre-Adjustment) [95% CI]	Median AUC (Post-Adjustment) [95% CI]	Median AUC Difference [95% CI]
Full Cohort	1890	0.77 [0.75, 0.79]	0.74 [0.72, 0.76]	-0.029 [-0.037,-0.023]
Biopsy-Naïve	1233	0.79 [0.75, 0.79]	0.73 [0.71, 0.75]	-0.041 [-0.048, -0.034]
Biopsy- Confirmed	1392	0.77 [0.75, 0.79]	0.74 [0.72, 0.76]	-0.029 [-0.036, -0.022]
Systematic Biopsy Only	343	0.77 [0.75, 0.79]	0.78 [0.76, 0.79]	0.007 [0.004, 0.010]
Targeted + Systematic Biopsy	709	0.77 [0.75, 0.79]	0.75 [0.73, 0.77]	-0.019 [-0.025, -0.014]

Each patient was matched with one in the reference population, stratified by grade group. A bootstrap sample size of 1000 was used. Adjustments were made using a linear transformation based on significant effects identified by each model, allowing comparison of AUC values pre- and post-adjustment. Adjusting for patient and acquisition effects did not improve csPCa detection using RSIrs_max_ (*p ≥ 0.05)*, suggesting the statistically significant effects on RSIrs_max_ in this cohort may be too small to affect the clinical utility of the imaging biomarker

Sensitivity analysis using the biopsy-naïve sub-cohort (*n* = 1,233) confirmed that the csPCa detection performance of RSIrs_max_ was not significantly biased by post-procedural changes in gland tissue. In this subset, the biomarker demonstrated an unadjusted AUC of 0.73 (95% CI: 0.71–0.75). In the biopsy-confirmed cohort (*n* = 1,392), where histopathology served as the sole reference standard, the unadjusted AUC was 0.77 (95% CI: [0.75,0.79]) and the adjusted AUC was 0.74 (95% CI: [0.72,0.76]). Across additional sub-cohorts, model estimates remained similar pre- and post-adjustment; AUC values ranged from 0.73 to 0.79 (Table 3), demonstrating stable diagnostic discrimination despite variation in pathologic confirmation methods.

Estimation of significant factor effects appeared stable with around 20 patients typically adequate for stable estimation of effects of an acquisition group from a different scanner manufacturer and RSI protocol (URMC) to the reference population (Supplementary Fig. 2).

## Discussion

In this multicenter study, we demonstrate that the primary driver of RSIrs variability is patient- rather than acquisition-related factors. Our variance component analysis showed that only 17.1% of total variability was attributable to scanner, protocol, or site effects, with a majority of variance associated with patient-level and residual factors. Specifically, Imaging Center (ICC = 0.09), Manufacturer Model (ICC = 0.03), and Protocol (ICC = 0.05). Furthermore, partial R^2^ was the highest for Grade Group compared to any other factor. Specifically, Grade Group 5 demonstrated the largest standardized effect size (+ 259.75) on RSIrs.

Overall, these findings indicate that while measurable systematic shifts exist across acquisition environments, they are modest relative to the underlying signal from csPCa. From a translational perspective, this suggests that RSIrs demonstrates sufficient intrinsic stability to function across heterogeneous 3T clinical platforms without mandatory post-hoc harmonization.

In addition, our results demonstrated that while these systematic acquisition-level shifts are statistically significant, they do not substantially affect csPCa detection performance. The modest attenuation in AUC following covariate adjustment likely reflects the unintentional removal of useful correlation between RSIrs and csPCa. Importantly, the magnitude of change was small (ΔAUC = 0.03, with overlapping confidence intervals between unadjusted and adjusted AUC), and center-level analyses demonstrated stable csPCa detection performance across institutions (AUC range: 0.73–0.81), except for UCSF (AUC: 0.58 [0.37, 0.78], *n* = 43) whose patient case-mix likely reflected a higher proportion of diagnostically challenging cases, as evidenced by the large confidence intervals. Regardless, these findings indicate that while measurable acquisition-level effects exist, they exert limited influence on RSIrs relative to the presence of csPCa. Our results suggest adjustment may be unnecessary, and RSIrs can be used effectively as a biomarker without major concern that other patient-level factors including age, self-reported race or ethnicity, prostate volume, or use of 5-ARIs will invalidate quantitative results. Previous work has shown that RSIrs_max_ performs comparably to expert PI-RADS interpretation [4, 6], and we find here that adjusting for potential patient- and acquisition-level bias did not significantly change its csPCa detection performance, even with some effects exhibiting quantitative overlap with presence of GG2 cancer.

Likewise, our analysis of systematic effects from different scanners and acquisition protocol parameters suggests that the utility of RSIrs as a biomarker is reproducible across a range of centers, scanners, vendors, and acquisition protocols regardless of significant systematic changes in RSI due to acquisition effects. These findings are consistent with the strong performance of RSIrs for csPCa detection in a recent study pooling heterogeneous data, although this study itself does not explore a comparison of RSIrs csPCa detection performance to the clinical standard PIRADS or ADC [6]. Here, we also demonstrate that the strongest factor impacting RSIrs_max_ in multivariable models is Grade Group, which is precisely the primary goal in developing a quantitative biomarker for csPCa.

The associations observed between RSIrs and patient-level covariates such as prostate volume and age are consistent with known benign microstructural changes in the prostate. Increasing prostate volume, often driven by benign prostatic hyperplasia (BPH), is characterized by expansion of fibromuscular stroma and extracellular matrix, which may increase the relative contribution of hindered extracellular diffusion and reduce the proportional restricted intracellular signal. Moreover, a larger gland has more voxels, giving a greater probability of finding a single high value (maximum) by chance. Aging is also associated with increased cell proliferation, resulting in reduced epithelial cellular density that could affect RSIrs. These processes may shift the balance among diffusion compartments modeled by RSI. Although these mechanisms were not directly measured in this study, accounting for age and prostate volume in multivariable analyses allows us to partially account for structural variation in the prostate when estimating the association between RSIrs and csPCa. Their systematic effect on RSI, on average, are not significant in the context of detecting csPCa.

Systematic biases can arise in laboratory values and biomarkers because of variation in the specific lab array, and any number of patient factors [27, 28]. Measuring these potential biases is key to ensuring accurate utility of any biomarker. The small effects observed in our study are reassuring, as are the results of the sample-size analysis, which suggest 20 patients can be adequate to estimate systematic deviation of a new population or new factor from a reference population. Overall, this study demonstrates the robustness of RSIrs as a reproducible biomarker.Prostate mpMRI has wide variation in performance in terms of positive predictive value (PPV) [29]. Quantitative biomarker development can improve reliability and reproducibility and is a stated priority of the NCI, NIBIB, RSNA, etc. [30–32]. ADC is the quantitative marker currently used in mpMRI interpretation, but it performs poorly as an objective biomarker in the absence of expert-defined suspicious lesions [4, 6, 30]. RSI and several other proposed advanced diffusion MRI models attempt to better explain the biophysical complexity of prostate tissue microstructure than ADC [7, 33, 34]. Each has shown improvements over conventional MRI, with a recent clinical trial finding a derived quantitative biomarker from Vascular, Extracellular, and Restricted Diffusion for Cytometry in Tumor (VERDICT) MRI, the fractional intracellular volume (FIC), as a superior classifier of csPCa [34]. A multicenter trial is also ongoing to evaluate the impact of RSIrs on accuracy of biopsy decisions by expert and non-expert radiologists (NCT06579417) [35, 36].

The evaluation of RSIrs robustness in this study aligns itself with established translational frameworks for quantitative imaging biomarkers into clinical workflow. O’Connor et al. emphasize that technical validation (i.e., repeatability and multicenter reproducibility) is a prerequisite for clinical integration of quantitative metrics [2]. By explicitly quantifying variance components across institutions, scanner platforms, and protocols, our study addresses key elements of this technical validation phase. Our findings are also consistent with consensus guidance from the Quantitative Imaging Biomarkers Alliance (QIBA), which highlights the importance of controlling acquisition variability in prostate diffusion imaging. Prior QIBA reports have documented substantial repeatability limits for conventional apparent diffusion coefficient (ADC) measurements in the prostate, with repeatability coefficients approaching 47% under certain conditions [3]. Direct repeatability testing of RSIrs is underway in a prospective, single-center study (NCT04992728) [37]. While direct repeatability comparisons were not performed in this study, the relatively modest proportion of variance attributable to acquisition-related factors suggests that RSIrs demonstrates stability within the tested multicenter environment. More broadly, recent multicenter investigations have demonstrated that RSIrs can maintain clinically useful positive predictive value for csPCa and Grade Group despite site heterogeneity [6]. Together, these findings support the feasibility of implementing RSIrs within contemporary multicenter clinical workflows.

From a deployment perspective, these findings suggest that RSIrs can be implemented across contemporary 3T GE and Siemens platforms. csPCa detection performance remained stable across centers, indicating preservation of csPCa signal despite measurable systematic shifts in raw RSIrs values. However, we recommend that institutions adopting RSIrs confirm that local RSIrs distributions fall within the variance and AUC ranges observed in this study as site-specific patient case mix may alter fixed decision thresholds. Rojo Domingo et al. determined specific RSIrs ranges for prediction thresholds that are a helpful guide for this purpose [6]. If local values fall substantially outside these ranges due to protocol deviations or atypical patient case-mix, threshold adjustment may be warranted.

Limitations of this study included reliance on interpretation with PI-RADS and on biopsy results, both of which are subject to inter-reader variability, though our approach mirrors the reality of clinical practice.

In addition, we used a composite reference standard in which low-risk patients (PI-RADS 1–2 and PSAD ≤ 0.15) were classified as non-csPCa without biopsy confirmation. While this introduces the potential for verification bias, this approach reflects current clinical practice, as biopsy is frequently deferred in such patients. The combination of negative mpMRI and low PSA density has demonstrated high negative predictive value (> 95%) for Grade Group ≥ 2 disease in similar populations [38, 39]. Any misclassification is expected to be minimal. Importantly, sensitivity analyses restricted to biopsy-confirmed cases demonstrated comparable model estimates and csPCa detection performance, supporting the robustness of our findings and suggesting that the inclusion of these patients as non-csPCa did not significantly alter conclusions regarding RSIrs technical robustness or discrimination performance.

Furthermore, multicenter data collection introduces potential confounding factors due to differences in patient case-mix, disease prevalence, biopsy practices, and demographic composition across institutions. Such differences can create apparent site effects that could affect csPCa detection performance of RSIrs. To mitigate this, we incorporated patient-level covariates (age, prostate volume, race/ethnicity, Grade Group) and modeled imaging center as a random intercept, allowing separation of patient-level and acquisition-level (i.e., institution-level) effects. However, unobserved factors such as differences in biopsy sampling density due to institutional guidelines may persist when calculating RSIrs.

It is also possible that non-linear modeling of patient or acquisition factors could be more effective than linear models for assessing systematic effects. On the other hand, performance for csPCa detection with RSIrs is already sufficient for clinical utility with the implementation of voxel-wise RSIrs overlays providing benefit for contouring lesions without expert radiologists [12]. We note that some of the patient factors are found in only a minority of patients analyzed here such as 5-ARI usage or Black race. Larger studies may prove informative, but it is reassuring that the sample-size analysis demonstrates a plateau at small sample sizes (< 30).

These findings primarily apply to the study cohort evaluated here, which includes 3T MRI systems (GE and Siemens platforms) using contemporary multiparametric prostate MRI protocols in men undergoing evaluation for suspected prostate cancer. Extrapolation to 1.5T systems, substantially different acquisition schemes, or alternative post-processing pipelines requires further validation and is not recommended.

The systematic effects measured here represent correlations and may not imply causality; patients from a given imaging center may also simply differ from other populations in ways not measurable in the present work. For acquisition factors, it is possible to scan the same patient with both approaches [20]. For most patient factors (age, race, prostate volume, 5-ARI use), though, re-scanning without the factor is not a feasible strategy.

In conclusion, within the population explored in this multicenter study, the RSI restriction score (RSIrs) demonstrated measurable robustness and stable csPCa detection performance. Variability attributable to center, scanner model, and protocol was modest relative to csPCa signal, and detection performance remained consistent across participating institutions. Accordingly, RSIrs appears suitable for deployment within acquisition environments comparable to those studied here.

## Supplementary Information

Below is the link to the electronic supplementary material.

Supplementary Material 1

## Data Availability

All data and code used in this study is available upon reasonable request. Please send a request to the corresponding author Dr. Tyler Seibert at [tseibert@health.ucsd.edu](mailto: tseibert@health.ucsd.edu) or the primary author Deondre Do at [d2do@health.ucsd.edu](mailto: d2do@health.ucsd.edu).
