# Supplementary material for: Systematic effects of patient factors and scanner/protocol factors on a Restriction Spectrum Imaging (RSI) quantitative MRI biomarker for prostate cancer
Source: Cancer Imaging. 2026 Apr 16;26:70. doi: 10.1186/s40644-026-01032-w (PMC13202994; doi:10.1186/s40644-026-01032-w)
Supplement: Supplementary file 1 — Supplementary Material 1 [file 40644_2026_1032_MOESM1_ESM.docx]

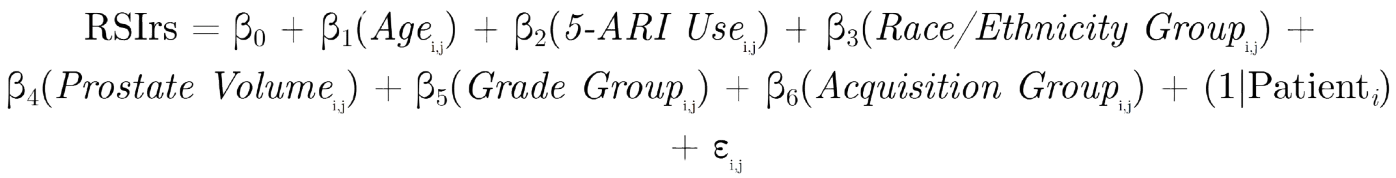


Supplementary Equation 1. Linear mixed effects model formula to predict RSIrs at the voxel level based on patient and acquisition factors for all prostate voxels in patients without benign csPCa. Absence of csPCa in this cohort was determined by either a negative biopsy (benign results or GG1 only) or non-suspicious mpMRI (PI-RADS 1 or 2). To minimize the possibility of occult csPCa affecting model estimation, we additionally excluded any patients without known csPCa if prostate-specific antigen density (PSAD) was ≥0.15. As each prostate contributes many voxels, we included patient case as a random effect in the mixed effects model to account for repeated measures. Models was fit using fitlme() in MATLAB^25^. Β_0_, β_1_, ..., β_5_, β_6_ denotes the respective predictor coefficient estimates, *I* represents the *i-*th patient, *j* represents the *j-*th voxel for the *i*-th patient, (1|Patient) represents the random effect term and ε represents the vector of residual error terms.

|  |  |  |
| --- | --- | --- |
| **Institution** | **Scanner Models** | **Number of Scanners** |
| UCSD CTIPM | GE Healthcare Discovery MR750,  GE Healthcare Signa Premier | 4 |
| UCSD Health | GE Healthcare Discovery MR750,  GE Healthcare Signa Premier | 4 |
| URMC | SIEMENS Magnetom Skyra | 2 |
| MGH | GE Healthcare Signa Premier | 1 |
| UCSF | GE Healthcare Signa Premier | 2 |
| Cambridge | GE Healthcare Discovery MR750 | 1 |
| UTHSCSA | SIEMENS Magnetom Skyra,  SIEMENS Magnetom Trio | 3 |
|  |  |  |
|  |  |  |
| **Cambridge** | **DWI** | **T2-weighted** |
| Pulse sequence | Diffusion-weighted EPI | Fast Spin Echo (FSE) |
| TR (ms) | 4500 | 3130 |
| TE (ms) | 59 | 98 |
| Voxel Size (mm) | 2.29 x 2.29 x 4 | 0.4 x 0.7 x 3 |
| *b-*values (s/mm^2) [number of samples] | 0[1], 500[6], 1000[6], 2000[12] | N/A |
| Field Strength (T) | 3 | 3 |
| Scanner Manufacturer/Model | GE Healthcare/Discovery MR750 | GE Healthcare/Discovery MR750 |
| **MGH** | **DWI** | **T2-weighted** |
| Pulse sequence | Diffusion-weighted EPI | Fast Spin Echo (FSE) |
| TR (ms) | 4500 | 3937 |
| TE (ms) | 59 | 169 |
| Voxel Size | 2.5 x 2.5 x 6 | 0.44 x 0.71 x 3 |
| *b*-values (s/mm^2^)  [number of samples] | 0[1],500[8],1000[8],2000[16] | N/A |
| Field Strength (T) | 3 | 3 |
| Scanner Manufacturer/Model | GE Healthcare/Signa Premier | GE Healthcare/Signa Premier |
| **UCSD CTIPM_Discovery1/UCSDH_Discovery1** | **DWI** | **T2-weighted** |
| Pulse sequence | Diffusion-weighted EPI | Fast Spin Echo (FSE) |
| TR (ms) | 4500 | 7000 |
| TE (ms) | 69 | 100 |
| Voxel Size (mm) | 2.5 x 2.5 x 6 | 0.75 x 0.75 x 3 |
| *b-*values (s/mm^2) [number of samples] | 0[1], 500[6], 1000[6], 2000[12] | N/A |
| Field Strength (T) | 3 | 3 |
| Scanner Manufacturer/Model | GE Healthcare/Discovery MR750 | GE Healthcare/Discovery MR750 |
| **UCSD CTIPM_Discovery2** | **DWI** | **T2-weighted** |
| Pulse sequence | Diffusion-weighted EPI | Fast Spin Echo (FSE) |
| TR (ms) | 4500 | 6230 |
| TE (ms) | 76 | 98 |
| Voxel Size (mm) | 2.5 x 2.08 x 3 | 0.75 x 0.75 x 3 |
| *b*-values (s/mm^2^)  [number of samples] | 0 [1], 50[6], 800[6], 1500[12], 3000 [18] | N/A |
| Field Strength (T) | 3 | 3 |
| Scanner Manufacturer/Model | GE Healthcare/Discovery MR750 | GE Healthcare/Discovery MR750 |
| **UCSDH_Premier** | **DWI** | **T2-weighted** |
| Pulse sequence | Diffusion-weighted EPI | Fast Spin Echo (FSE) |
| TR (ms) | 4500 | 3423 |
| TE (ms) | 59 | 170 |
| Voxel Size (mm) | 1.9656 x 2 x 4 | 0.45 x 0.7 x 3 |
| *b*-values (s/mm^2^)  [number of samples] | 0[1], 500[6], 1000[6], 2000[12] | N/A |
| Field Strength (T) | 3 | 3 |
| Scanner Manufacturer/Model | GE Healthcare/Signa Premier | GE Healthcare/Signa Premier |
| **UCSF/UCSD CTIPM_Premier** | **DWI** | **T2-weighted** |
| Pulse sequence | Diffusion-weighted EPI | Fast Spin Echo (FSE) |
| TR (ms) | 4500 | 2964 |
| TE (ms) | 75 | 150 |
| Voxel Size (mm) | 2.5 x 2.5 x 3 | 0.43 x 0.43 x 3 |
| *b*-values (s/mm^2^)  [number of samples] | 0[5], 100[6], 800[12], 1400[12], 2500[18] | N/A |
| Field Strength (T) | 3 | 3 |
| Scanner Manufacturer/Model | GE Healthcare/Signa Premier | GE Healthcare/Signa Premier |
| **URMC** | **DWI** | **T2-weighted** |
| Pulse sequence | Diffusion-weighted EPI | Fast Spin Echo (FSE) |
| TR (ms) | 3800 | 4800 |
| TE (ms) | 85 | 104 |
| Voxel Size (mm) | 1 x 1 x 4 | 0.47 x 0.49 x 3 |
| *b*-values (s/mm^2^)  [number of samples] | 0[1], 500[6], 1000[6], 2000[6] | N/A |
| Field Strength (T) | 3 | 3 |
| Scanner Manufacturer/Model | SIEMENS Healthcare/Magnetom Skyra | SIEMENS Healthcare/Magnetom Skyra |
| **UTSA_Skyra** | **DWI** | **T2-weighted** |
| Pulse sequence | Diffusion-weighted EPI | Fast Spin Echo (FSE) |
| TR (ms) | 6300 | 4710 |
| TE (ms) | 105 | 100 |
| Voxel Size (mm) | 1 x 1 x 4 | 0.75 x 0.56 x 3 |
| *b*-values (s/mm^2^)  [number of samples] | 0[1], 500[6], 1000[18], 2000[42] | N/A |
| Field Strength (T) | 3 | 3 |
| Scanner Manufacturer/Model | SIEMENS Healthcare/Magnetom Skyra | SIEMENS Healthcare/Magnetom Skyra |
| **UTSA_Trio** | **DWI** | **T2-weighted** |
| Pulse sequence | Diffusion-weighted EPI | Fast Spin Echo (FSE) |
| TR (ms) | 98 | 1800 |
| TE (ms) | 5500 | 203 |
| Voxel Size (mm) | 2.3438 x 2.3438 x 3 | 0.68 x 0.68 x 1.5 |
| *b*-values (s/mm^2^)  [number of samples] | 0[1], 500[30], 1000[30], 2000[30] | N/A |
| Field Strength (T) | 3 | 3 |
| Scanner Manufacturer/Model | SIEMENS Healthcare/Magnetom Trio | SIEMENS Healthcare/Magnetom Trio |

Supplementary Table 1. Detailed Breakdown of MRI parameters for all acquisition groups. *N/A* signifies the parameter was not relevant for the acquisition. Parameters may differ between patient MRI scans. Abbreviations: TR (Repetition Time), TE (Echo Time), FSE (Fast Spin Echo), EPI (Echo Planar Imaging), Cambridge ( University of Cambridge), MGH (Harvard University’s Massachusetts General Hospital), UCSD[H] (University of California San Diego [Health]), CTIPM (Center for Translational Imaging and Precision Medicine), UCSF (University of California San Francisco), URMC (University of Rochester Medical Center), UTHSCSA (University of Texas Health Sciences Center San Antonio).

|  |  |
| --- | --- |
| **Model** | All Patients (RSIrs_99_) |
| **Formula** |  |
|  | RSIrs_99_ ~ 5-ARI use + Age + Prostate volume  + Race/Ethnicity group + Grade group + Acquisition group |
| **Age** [reference = mean, 69 years old] |  |
|  | **1.07***  **[0.04, 2.11]** |
| **Prostate Volume** [reference = mean, 60 mL] |  |
|  | **-0.80*****  **[-1.08, -0.52]** |
| **5-ARI Use** [reference = Not used] |  |
| On Use within 6 months of MRI | 29.69  [-15.78, 75.16] |
| **Race/Ethnicity Group** [reference = White Non-Hispanic] |  |
| Asian | 14.25  [-19.70, 48.20] |
| Black | 6.51  [-22.25, 35.26] |
| White Hispanic | -13.34  [-46.76, 20.07] |
| **Grade Group** [reference = Benign] |  |
| 1 | 8.43  [-16.11, 33.01] |
| 2 | **31.77****  **[9.18, 54.36]** |
| 3 | **56.51*****  **[30.12, 82.91]** |
| 4 | **112.78*****  **[72.451, 153.10]** |
| 5 | **182.8*****  **[149.12, 216.48]** |
| **Acquisition Group** [reference = UCSD CTIPM_Discovery1] |  |
| MGH | 43.51  [-24.12, 111.14] |
| UCSD CTIPM_Discovery2_ | **-68.54*****  **[-98.85, -38.23]** |
| UCSDH_Premier_ | 29.12  [-1.99, 60.22] |
| UCSF/UCSD CTIPM_Premier_ | **-41.67****  **[-72.9, -10.43]** |
| URMC | **33.33****  **[12.39, 54.28]** |
| UTSA_Skyra_ | -39.66  [-83.89, 4.57] |
| UTSA_Trio_ | **-45.05****  **[-77.01, -13.07]** |

Supplementary Table 2. All predictors and their estimated effects on the RSIrs biomarker identified using multiple linear regression (99th percentile RSIrs) and linear mixed effects modeling (total RSIrs distribution). These predictors included 5-ARI (current 5-ARI usage or usage <6 months before MRI), age, prostate volume, race/ethnicity, grade group, and acquisition group. Coefficient estimates [95% confidence interval] are reported for each significant effect, which provide insight into the impact of these variables on RSIrs. *N/A* signifies there were no representative patients of that category included in the analysis. Significant predictors: * (*p < 0.05), ** (p < 0.01), *** (p < 0.001)*

| **Model** | Benign [no csPCa] (Voxel-wise) |
| --- | --- |
| **Formula** |  |
|  | RSIrs ~ 5-ARI use + Age + Prostate volume  + Race/Ethnicity group + Grade group + Acquisition group + (1\|Patient) |
| **Age** [reference = mean, 69 years old] |  |
|  | **0.36****  **[0.11, 0.61]** |
| **Prostate Volume** | [reference = mean, 74 mL] |
|  | -0.04  [-0.09, 0.02] |
| **5-ARI Use** [reference = Not used] |  |
| On Use within 6 months of MRI | 2.75  [-8.04, 13.54] |
| **Race/Ethnicity Group** [reference = White Non-Hispanic] |  |
| Asian | -1.74  [-11.59, 8.12] |
| Black | 2.55  [-3.78, 8.89] |
| White Hispanic | -0.74  [-7.85, 6.37] |
| **Grade Group** [reference = Benign] |  |
| 1 | -1.57  [-5.34, 2.19] |
| 2 | N/A |
| 3 | N/A |
| 4 | N/A |
| 5 | N/A |
| **Acquisition Group** [reference = UCSD CTIPM_Discovery1_/UCSDH_Discovery_] |  |
| MGH | N/A |
| UCSD CTIPM_Discovery2_ | **-12.84*****  **[-19.31, -6.36]** |
| UCSDH_Premier_ | **19.5*****  **[12.88, 26.17]** |
| UCSF/UCSD CTIPM_Premier_ | **-15.12***  **[-28.64, -1.60]** |
| URMC | **13.60*****  **[9.13, 18.07]** |
| UTSA_Skyra_ | **-14.88****  **[-25.72, -4.04]** |
| UTSA_Trio_ | **-13.4*****  **[-19.73, -7.07]** |

Supplementary Table 3. All predictors and their estimated effects on the RSIrs biomarker identified using linear mixed effects modeling. These predictors included 5-ARI (current 5-ARI usage or usage <6 months before MRI), age, prostate volume, race/ethnicity, grade group, and acquisition group. Linear mixed effects modeling considered multiple voxels within the same patient. Coefficient estimates [95% confidence interval] are reported for each significant effect, which provide insight into the impact of these variables on RSIrs. *N/A* signifies there were no representative patients of that category included in the analysis. Significant predictors: * (*p < 0.05), ** (p < 0.01), *** (p < 0.001)*

| **Model used for Adjustments** | **Median AUC (Pre-Adjustment)** | **Median AUC (Post-Adjustment)** | **Median AUC Difference** |
| --- | --- | --- | --- |
| Voxel-wise model in patients without csPCa | 0.77   [0.75, 0.79] | 0.77   [0.76, 0.79] | 0.005   [0.004, 0.006] |

Supplementary Table 4. Results from a 10,000-bootstrap analysis using a subgroup of patients with significant acquisition and patient effects on RSIrs_max_. Each patient was matched with one in the reference population, stratified by grade group. A bootstrap sample size of 1000 was used. Adjustments were made using a linear transformation based on significant effects identified by each model, allowing comparison of AUC values pre- and post-adjustment. Adjusting for patient and acquisition effects did not improve csPCa detection using RSIrs_max_ (*p ≥ 0.05)*, suggesting the statistically significant effects on RSIrs_max_ in this cohort may be too small to affect the clinical utility of the imaging biomarker.

| **Model used for Adjustments** | **Median AUC (Pre-Adjustment)** | **Median AUC (Post-Adjustment)** | **Median AUC Difference** |
| --- | --- | --- | --- |
| Voxel-wise model in patients without csPCa | 0.76  [0.74, 0.78] | 0.77  [0.75, 0.79] | 0.007  [0.006, 0.009] |
| Patient-level model of RSIrs_max_ using all patients | 0.74  [0.72, 0.76] | 0.73  [0.70, 0.75] | -0.01  [-0.02, -0.005] |

Supplementary Table 5. Results from two 10,000-bootstrap analyses using subgroups of patients with significant acquisition and patient effects on RSIrs estimated with two methods, respectively. Each patient was matched with one in the reference population, stratified by grade group. A bootstrap sample size of 10,000 was used. Adjustments were made using a linear transformation based on significant acquisition and patient effects identified by two different estimation methods, allowing comparison of AUC values pre- and post-adjustment. This reflects the impact of both acquisition and patient effects on RSIrs.

| **Random Effect** | **Variance Estimate** | **Standard Deviation [95% CI]** | **% of Total Variance**  **(ICC)** |
| --- | --- | --- | --- |
| Imaging Center | 3938 | 62.76 [32.12, 122.63] | 9.23% |
| Scanner Manufacturer/Model | 1467 | 38.30 [12.10, 121.21] | 3.44% |
| Acquisition Protocol | 2084 | 45.65 [23.61, 88.26] | 4.88% |
| Residual (Patient-Level/Unmodeled) | 35180 | - | 82.45% |
| Total Variance | 42669 | - | 100% |

Supplementary Table 6. Random effects parameters and Intraclass Correlation Coefficients (ICC) for the multi-center RSIrs model. Variance components were estimated using a linear mixed-effects model. The ICC represents the percentage of total variance explained by each technical factor. 95% confidence intervals for standard deviations were calculated during linear modeling.

| **Institution** | **N (Total)** | **N (csPCa)** | **Prevalence (%)** | **Median**  **Unadjusted AUC [95% CI]** |
| --- | --- | --- | --- | --- |
| UC San Diego Health | 692 | 252 | 36.41618497 | 0.79 [0.75, 0.83] |
| UC San Diego CTIPM | 688 | 306 | 44.47674419 | 0.8 [0.76, 0.83] |
| Harvard University Massachusetts General Hospital | 64 | 28 | 43.75 | 0.81 [0.70, 0.91] |
| University of Rochester Medical Center | 251 | 107 | 42.62948207 | 0.73 [0.66, 0.80] |
| UC San Francisco | 43 | 26 | 60.46511628 | 0.58 [0.37, 0.78] |
| UT Health Sciences Center San Antonio | 147 | 40 | 27.21088435 | 0.79 [0.69, 0.88] |
| University of Cambridge | 5 | 1 | 20 | 0.5 [0, 1] |
| All Sites Combined | 1890 | 760 | 40.21164021 | 0.77 [0.75, 0.79] |

Supplementary Table 7: Comparison of diagnostic performance across participating institutions. Total patients, positive cases, and disease prevalence are summarized alongside median unadjusted AUC values and 95% confidence intervals. These data characterize the institutional diversity of the study population and the consistency of RSIrs across clinical settings.

| **Term** | **F-Statistic** | ***df1*** | ***df2*** | p-value | **Partial R^2^** |
| --- | --- | --- | --- | --- | --- |
| Intercept | 8.63 | 1 | 1031 | 0.003 | 0.008 |
| Age | 5.09 | 1 | 1031 | 0.024 | 0.005 |
| Race/Ethnicity | 0.61 | 3 | 1031 | 0.606 | 0.002 |
| Prostate Volume | 14.81 | 1 | 1031 | < 0.001 | 0.014 |
| Grade Group | 25.82 | 5 | 1031 | < 0.001 | 0.111 |
| Acquisition Group | 5.2 | 7 | 1031 | < 0.001 | 0.034 |
| 5-ARI Medication Use | 1.08 | 1 | 1031 | 0.3 | 0.001 |

Supplementary Table 8: ANOVA for Global Fixed Effects on RSIrs_max_​. ANOVA marginal tests were performed using the residual method for degrees of freedom (n = 1050 observations). Global Partial R^2^ values represent the proportion of unique variance accounted for by each term in the hierarchical linear mixed-effects model.


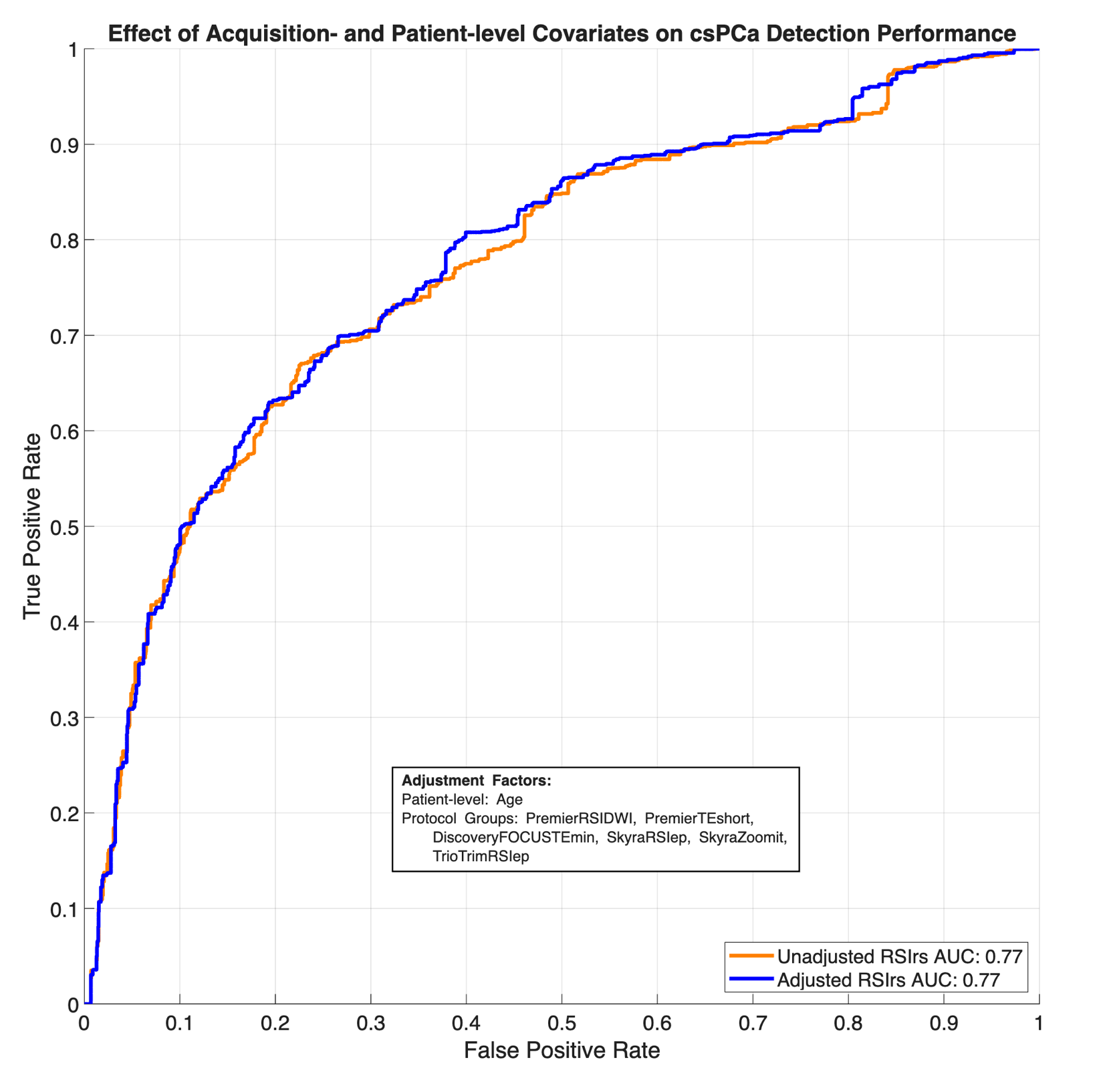


Supplementary Figure 1. ROC curves illustrate the detection performance of RSIrs_max_ for clinically significant prostate cancer (csPCa) pre-adjustment and post-adjustment using shifts estimated from patients without csPCa. Pre-adjustment RSIrs_max_ performance (orange line) is compared with post-adjustment performance (blue line) after pooling data from 10,000 bootstrap samples. The area under the curve (AUC) values are reported for each model, demonstrating the impact of acquisition and patient adjustments on the predictive accuracy of RSIrs_max_ for csPCa. Median pre-adjustment AUC was 0.77 [95% CI: 0.75-0.79] and median post-adjustment AUC was 0.77 [0.76-0.79]; median AUC difference was 0.005 [0.004, 0.006]. Adjustment for patient and acquisition effects does not significantly affect the AUC (*p* < 0.05).


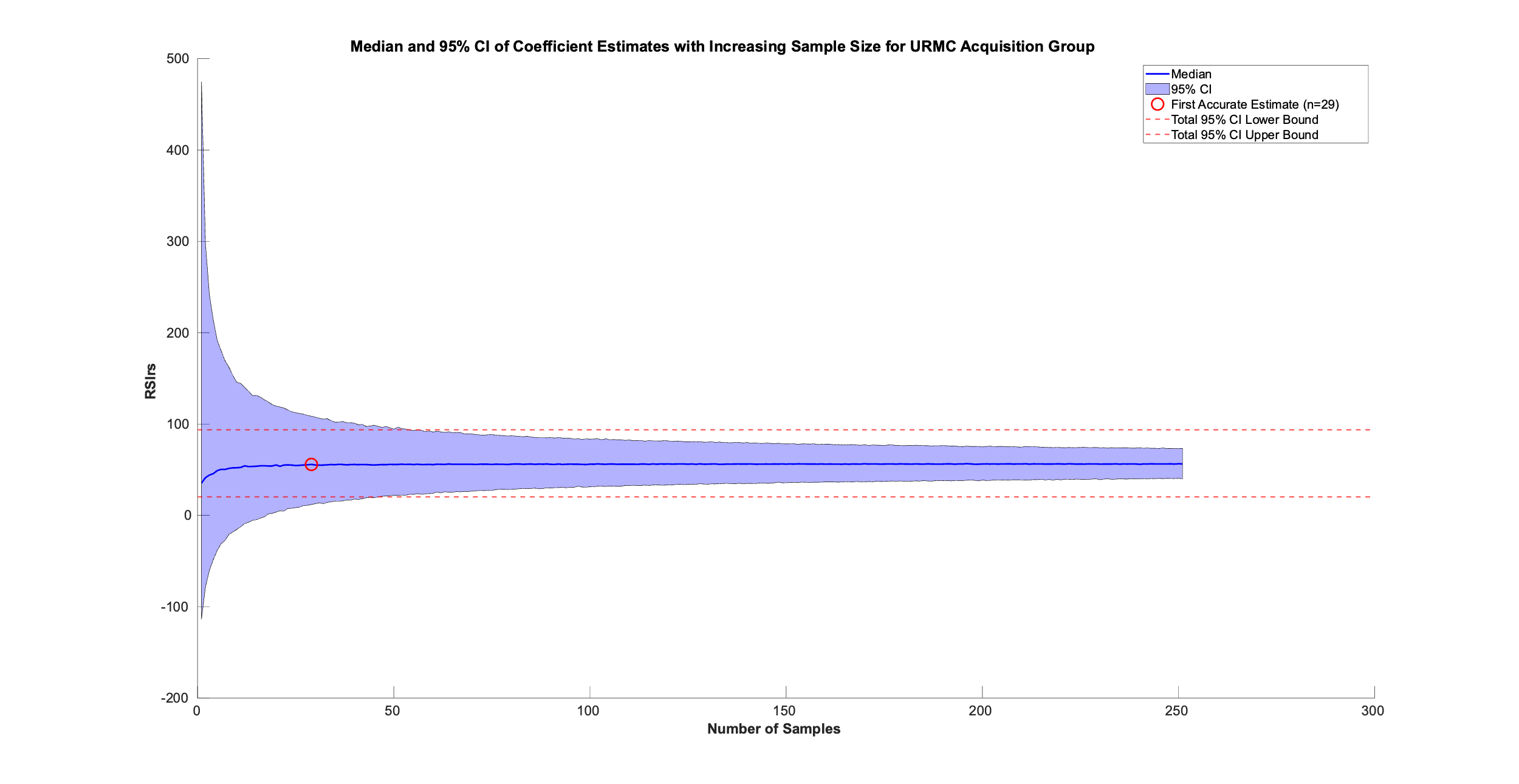
Supplementary Figure 2. Sample size estimation analysis for an acquisition group from University of Rochester Medical Center (URMC). In this analysis, 10,000 bootstrap samples ranging from n = 1:251 were taken. The line on the graph represents the median effect estimate for each set of 10,000 bootstrap samples (RSIrs), while the blue shading indicates the 95% Confidence Interval for each set of 10,000 bootstrap samples. The red dotted lines represent the mean 95% Confidence Interval of all sets of bootstrap samples. The red circle denotes the location of the minimum estimated sample size required to accurately reproduce the acquisition effects at this institution, defined as where the minimum bootstrap median coefficient estimate that is within 1% of the value observed for the total population (n=251). Accurate estimation of acquisition effects requires only 29 patients.


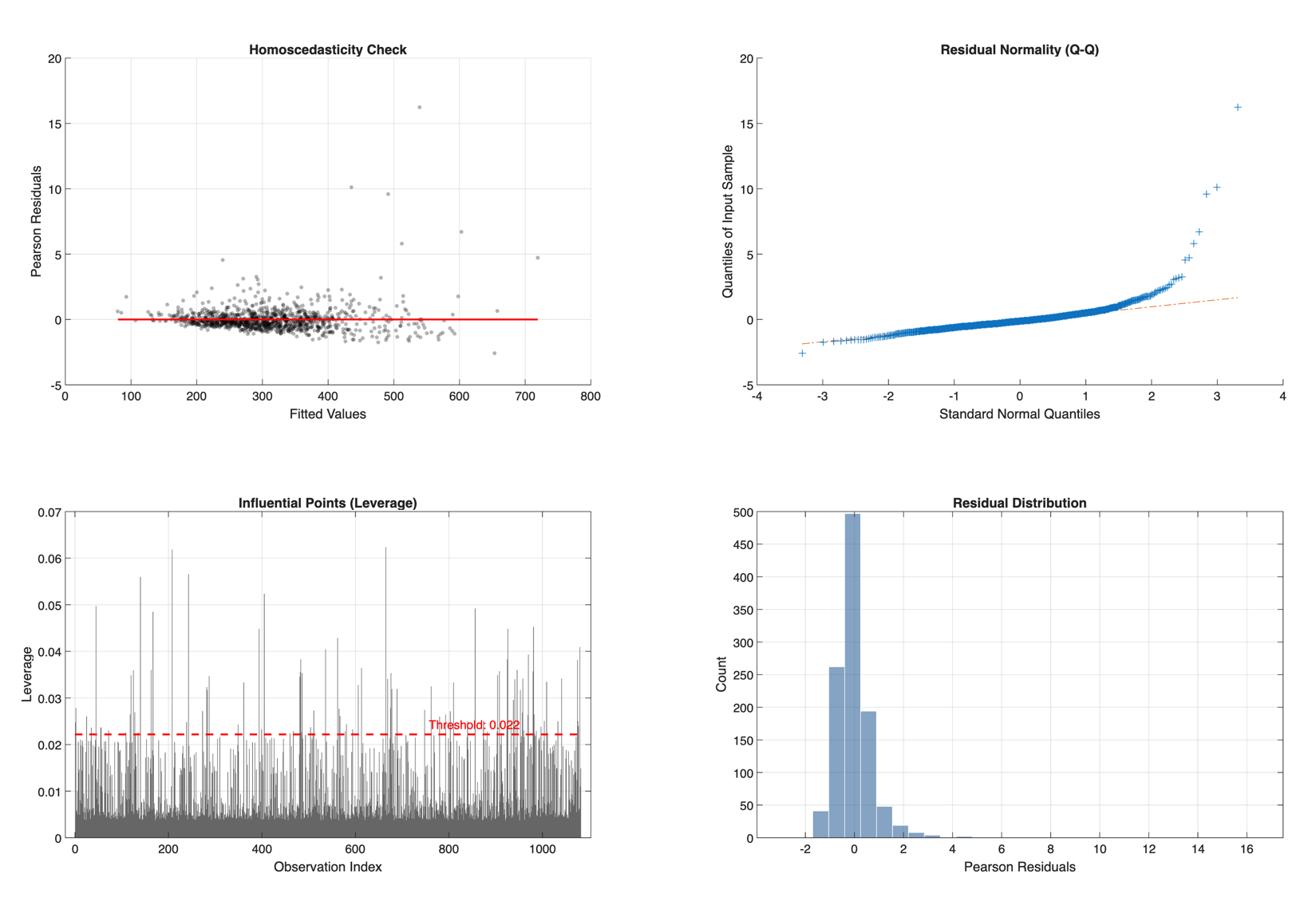


Supplementary Figure 3: Diagnostic assessment of the linear model for RSIrs. A. Homoscedasticity check plotting standardized Pearson residuals against fitted RSIrs_max_ values. The horizontal red line at the zero intercept indicates a lack of systematic bias and constant variance across the clinical range of the biomarker. B. Normal Quantile-Quantile (Q-Q) plot evaluating the distribution of model residuals. The alignment of observations with the reference line confirms the normality of error terms; the upward deviation in the right tail reflects the characteristically intense signal typical of high-grade csPCa (i.e., Grade Group 5). C. Leverage plot for the identification of influential observations. The dashed red line represents the conservative high-leverage threshold (*l* = 0.036). While 82 high-leverage points were identified, no observations were simultaneously high-outlier (Pearson residual > ∣ 3 ∣), confirming model stability. D. Frequency distribution of standardized Pearson residuals, demonstrating a centrally peaked distribution at zero, confirming the reliability of the global multicenter estimates.
